# A Dive Into Oliceridine and Its Novel Mechanism of Action

**DOI:** 10.7759/cureus.19076

**Published:** 2021-10-27

**Authors:** Anjali Shah, Reema Shah, Germin Fahim, Lindsay A Brust-Sisti

**Affiliations:** 1 Pharmacy, Rutgers University, Piscataway, USA; 2 Pharmacy, Monmouth Medical Center, Long Branch, USA; 3 Pharmacy, Jersey City Medical Center, Jersey City, USA

**Keywords:** analgesic combination, abdominoplasty, bunionectomy, postoperative morphine use, post operative pain management, oliceridine, opioid epidemic

## Abstract

The current state of the opioid epidemic has revealed the need of utilizing proper pain management, especially in the postoperative setting where there is overuse of potent analgesics. However, the adequate treatment of pain is necessary to reduce mortality and cost of burden while increasing recovery and improving quality of life. Treatment of pain can be difficult to standardize as the guidelines from the American Pain Society discuss the importance of tailoring treatment options based on a patient’s sensitivities and risk factors. An effective fast-acting analgesic with adequate potency and few adverse events is the key to alleviating acute pain. Oliceridine (Olinvyk^®^, Trevena Inc., Chesterbrook, USA) is a novel G protein-biased μ-opioid receptor agonist designed to decrease opioid-related adverse events (ORAEs) compared to conventional opioids. This article discusses oliceridine’s novel mechanism of action and current place in therapy. After a literature search on clinicaltrials.gov, three clinical trials were analyzed to understand the safety and efficacy of oliceridine. These trials demonstrated a comparable efficacy to morphine with a decreased risk for serious adverse events. However, further studies need to be conducted to evaluate the true safety impact of oliceridine compared to conventional opioids.

## Introduction and background

Pain is the unpleasant sensory and emotional experience associated with actual or potential tissue damage [1]. Pain can be classified as acute or chronic, each with different management options. Acute pain is characterized as being self-limiting, lasting less than three to six months with treatment focused on the use of analgesics. Postoperative pain is a type of acute pain occurring in patients after undergoing a procedure. There is a preconceived notion that acute pain will be relatively short-lived after a successful operation. However, evidence suggests that less than half of patients who undergo surgery report adequate postoperative pain relief [2]. Inadequate acute pain management can transition to chronic pain through neuronal plasticity and increase mortality, delay recovery, and increase hospital costs [3]. This paper will review oliceridine (Olinvyk®, Trevena Inc., Chesterbrook, USA), its novel mechanism of action, and its role in pain management, especially with the current state of the ongoing opioid epidemic. 

In 2012, about 313 million surgical operations were performed globally, while about 28 million inpatient surgical procedures and 48 million ambulatory surgeries were reported in 2006 and 2010, respectively [4]. Postoperative pain is preventable and treatable, but it is often inadequately managed as pain response and sensitivity are different in all patients and management requires an individualized approach. More than 80% of patients who undergo surgical procedures experience acute postoperative pain and approximately 75% of those with postoperative pain report the severity as moderate, severe, or extreme. 

The economic burden of postoperative pain is not easy to estimate, but with the number of patients undergoing surgery, the burden is large due to the direct costs in healthcare resources and the indirect costs from the reduction in patient function and productivity. This is furthered through the inadequate management of postoperative pain-causing extended hospitalization, increased morbidity, and mortality associated with chronic pain [1]. The cost of transitioning from acute to chronic pain in one patient can be up to $1 million and overall the burden of cost for chronic pain in the United States is $560-$635 billion, higher than the individual costs of heart disease, cancer, and diabetes [4]. Our knowledge about the increased risks associated with inadequate management is linked with increased utilization of potent analgesics, such as opioids. However, the prescribing of opioids to help manage postoperative pain is not appropriate for everyone. On the other side of the spectrum, the opioid crisis has led to increased healthcare costs and loss of productivity by $100 billion per year [5]. Finding new strategies to properly control pain will drastically decrease the cost burden on the healthcare system and efficiently allow us to utilize healthcare resources [6]. 

Current pain management options

Conventional parenteral opioids have a narrow therapeutic window and are associated with dose-limiting opioid-related adverse events (ORAEs) such as vomiting, nausea, and opioid-induced respiratory depression (OIRD) [7]. As expected, the risk of these ORAEs frequently increases in the elderly and patients with comorbidities, but studies have shown that the risk also increases in children and adolescents between the ages of 12 to 17 years [8]. Health care providers must determine the fine line between overuse and underuse of these drugs. Table 1 mentions the current pain management options utilized in the hospital setting for postoperative pain.

**Table 1:** Current Pain Management Options for Postoperative Pain IV, intravenous; MOA, mechanism of action; NSAIDs, non-steroidal anti-inflammatory drugs; COX-1, cyclooxygenase-1; COX-2, cyclooxygenase-2; CNS, central nervous system; PNS, peripheral nervous system; NMDA, N-methyl-d-aspartate

Analgesic Type	Advantages	Disadvantages
IV Opioids (i.e. morphine, hydromorphone, fentanyl)	MOA: bind to receptors in the central nervous system and peripheral tissues and modulate the effect of the nociceptors [9]	Side effects [9]: a) Significant: respiratory depression b) Common: nausea, vomiting, pruritus, reduction in bowel motility
	Rapid onset of action with peak effect occurring in 1-2 hours [9]	Requires special consideration of use in morbidly obese patients or chronic pain patients [9]
	Potent [9]	Need regular monitoring of respiration and oxygen saturation in postoperative patients [9]
IV Acetaminophen	MOA: not fully understood but shown to prevent prostaglandin production in the CNS and PNS to inhibit pain impulses [10]	Side effects: infection, phlebitis, and local irritation [11]
	Rapid and high plasma concentration achieved within five minutes [10]	Contraindications: hypersensitivity, severe hepatic impairment or severe active hepatic disease [12]
	Pain relief occurs within few minutes [10]	
	Easily passes through blood brain barrier and preferred in multimodal analgesia [10]	
IV NSAIDs (i.e. ibuprofen, naproxen, ketorolac)	MOA: inhibit prostaglandin production through COX-1 and COX-2 [13]	Side effects: increased risk of gastrointestinal bleeding, cardiotoxicity, hepatotoxicity, renal dysfunction, and drug induced asthma [13]
	Plasma half life of ketorolac: about 5.5 hours [14]	Contraindications/warnings: a) Avoid during pregnancy b) Patients with salicylate hypersensitivity or allergic reaction (urticaria, asthma, etc.) [15]
	Ketorolac is the most commonly used IV NSAID and reduces opioid consumption by 25-45% [13]	Ketorolac cannot be used more than five days [13]
IV Ketamine	MOA: noncompetitive NMDA receptor antagonist believed to have antihyperalgesic effects and reverse opioid tolerance; may also suppress pain transmission by limiting astrocyte and microglial activation [16]	Side effects: hallucinations, agitation, euphoria, dysphoria, anxiety, nausea, tachycardia, sedation, and dizziness [16]
	Half life: 10-15 minutes [17]	Contraindications: pregnancy, psychosis, uncontrolled hypertension, severe liver dysfunction [18]
	Can be used in patients in opioid-dependent or opioid tolerant patients [16]	

Oliceridine's mechanism of action

The United States Food and Drug Administration (FDA) granted approval to Travena Inc. for oliceridine on August 7, 2020 after following a rigorous review to evaluate the risk versus benefit as addressing the opioid epidemic is the FDA’s top priority [19]. Oliceridine is a full opioid agonist with selectivity towards the G protein pathway and is indicated in adults for the management of severe acute pain that requires an intravenous (IV) opioid analgesic after inadequate management with alternative treatments [20]. Conventional opioids, such as morphine, produce analgesia on the central μ-opioid receptors and lead to a cascade of post-receptor signaling events through the G protein pathways, leading to the potent analgesia and the β-arrestin pathway, which results in ORAEs. Due to its selectivity, oliceridine has shown decreased recruitment of β-arrestin and is expected to cause fewer ORAEs [7]. Oliceridine should be reserved for use in a controlled clinical setting in patients for whom alternative treatment options have not been tolerated, are not expected to be tolerated, have not provided adequate analgesia, or are not expected to provide adequate analgesia [19,20]. 

Methods

A search of oliceridine on clinicaltrials.gov, a public registry listing clinical trials, was conducted to obtain relevant clinical trials and review the safety and efficacy of the drug’s novel mechanism of action. The search was limited to “interventional studies (clinical trials)” and “studies with results.” Search results were restricted to 2013 to the current time frame to focus on the most current pain management research. Six relevant clinical trials were found. The summary of these trials is outlined in Table 2. The relevance of a trial in this review is based on the current indication of oliceridine. 

**Table 2:** Results From Oliceridine Literature Search *Terminated

Study Title	Trial Abbreviation	NCT Number	Phase Type	Year
Open Label Study to Evaluate the Safety of TRV130 in Patients with Acute Pain	ATHENA-1	NCT02656875	Phase III	12/2015 - 5/2017
Study of Olicerdine (TRV130) for the treatment of Moderate to Severe Acute Pain After Abdominoplasty	APOLLO-2	NCT02820324	Phase III	5/2016 - 12/2016
Study of Oliceridine (TRV130) for the Treatment of Moderate to Severe Acute Pain After Bunionectomy	APOLLO-1	NCT02815709	Phase III	5/2016 - 12/2016
A Pilot Study of TRV130 for the Treatment of Fracture Pain*		NCT02520297	Phase II	10/2015 - 10/2015
A Study of TRV130 for the Treatment of Pain After Abdominoplasty		NCT02335294	Phase II	12/2014 - 7/2015
A Study of TRV130 for the Treatment After Bunionectomy		NCT02100748	Phase II	4/2014 - 10/2014

## Review

ATHENA-1

The ATHENA-1 trial [7] was a phase 3, multi-center, open-label study between December 2015 to May 2017 that evaluated the safety and effectiveness of oliceridine in patients with moderate to severe acute pain, warranting the use of a parenteral opioid. A total of 768 adult post-operative surgical and non-surgical patients with painful medical conditions reporting a pain score of ≥ 4 on an 11-point numerical rating scale (NRS) received oliceridine and were included in the safety and efficacy analysis. 

Enrolled patients were treated with IV oliceridine via clinician-administered bolus dosing and/or patient-controlled analgesia (PCA). Patients treated with IV oliceridine were given a loading dose followed by a supplemental dose if needed. Subsequent doses were administered on an as-needed basis. In patients that were also given PCA, they were provided with a loading dose and a demand dose using a six-minute lockout interval. If clinically indicated, supplemental doses were allowed as needed as early as 15 minutes after the initial dose. The duration of treatment was determined by the clinical need for parenteral opioid therapy, but the maximal duration of oliceridine treatment was limited to 14 days. There was no restriction on the prior use of opioids or non-opioid analgesics, perioperative use of local anesthetics, and epidural and intrathecal opioids, prior or concomitant use of anxiolytics, sedatives, and hypnotics, or concomitant non-opioid analgesics as part of a multimodal analgesic approach. However, patients were not allowed to use other parenteral or oral opioids during treatment with oliceridine. 

Oliceridine was found to be associated with a potent analgesic effect and rapid onset of action. Lack of efficacy leading to discontinuation was reported in less than 5% of patients. The mean NRS pain score at baseline was 6.3 ± 2.1. Oliceridine showed a rapid reduction in pain intensity. The mean change from baseline was -2.2 ± 2.3 at 30 minutes after the first dose. Oliceridine showed maintenance of pain reduction. The mean change from baseline at the end of treatment was -3.1 ± 3.1. 

Oliceridine demonstrated a favorable safety and tolerability profile, especially for those at risk for opioid-related complications. Sixty-four percent of all patients reported at least one adverse event (AE) during the study. Most AEs were mild to moderate, but severe AEs occurred in 2% of patients. The intensity of the AEs was similar across all cumulative dose groups. The most commonly reported AEs were nausea (31%), constipation (11%), and vomiting (11%) and incidence was dose-dependent. There were no differences in AEs in patients receiving pain management as bolus or PCA. Serious AEs were observed in 3% of patients with most of these AEs being attributed to complications of surgery, secondary to an underlying medical condition, or secondary to opioid therapy. Three patients experienced serious AEs “possibly” related to oliceridine which were postoperative ileus, respiratory depression, and hepatic/renal failure. There was decreased sedation in the oliceridine dosing groups. A total of 97% of patients reported “none to mild” withdrawal symptoms. 

APOLLO-1

The APOLLO-1 trial [21] was a phase 3, randomized, double-blind, placebo- and active-controlled study between May 2016 to December 2016 that measured safety, effectiveness, and tolerability of oliceridine compared to the placebo and morphine regimens. A total of 418 adults were eligible and scheduled to undergo primary, unilateral, first-metatarsal bunionectomy with osteotomy and internal fixation. Post-surgery, the patients were enrolled into the clinical trial and only were given study medication if they reported at least moderate pain, which was measured on a categorical scale (none, mild, moderate, and severe) and on an 11-point NRS (NRS>4) within nine hours after discontinuation of regional perineural anesthesia. 

The enrolled patients were randomized into one of the five treatment regimens, placebo, oliceridine 0.1 mg, oliceridine 0.35 mg, oliceridine 0.5 mg, or morphine 1 mg. The patients received a clinician-administered fixed IV loading dose followed by demand doses administered through a PCA. The PCA doses were administered 10 minutes after the loading dose. Patients could receive protocol-specified open-label rescue pain medication with etodolac 200 mg every six hours as needed. These patients continued with the study medication unless both were inadequate. Additionally, prophylactic antiemetics and prophylactic supplemental oxygen were not permitted during the randomized treatment period. 

The proportion of treatment responders through 48 hours was statistically superior for all of the oliceridine treatment regimens compared to placebo. Based on the incidence of AEs and comparable efficacy to morphine, oliceridine regimen 0.35 mg appears to be the optimal dose to achieve the adequate analgesic effect.

Amongst the three oliceridine treatment groups there were no statistical differences on the composite measure of respiratory safety burden (RSB) compared to the morphine group. However, when looking at these treatment groups individually, patients receiving 0.1 mg and 0.35 mg oliceridine experienced significantly lower respiratory safety compared to morphine whereas patients receiving 0.5 mg did not show a difference compared to morphine. Amongst the oliceridine treatment groups, the gastrointestinal (GI) related AEs increased in a dose-dependent manner. The most common AEs were comparable between oliceridine and conventional opioids; these included nausea, vomiting, headache, dizziness, constipation, somnolence or sedation, pruritus, and dry mouth. No patient in the oliceridine treatment groups experienced a serious AE, and only a few reported experiencing an AE that was classified as severe. Compared to morphine-treated patients (7.9%), there were fewer oliceridine patients (2.6%) who discontinued treatment due to an AE. No deaths were reported during the study.

APOLLO-2

The APOLLO-2 trial [22] was a phase 3, multicenter, randomized, double-blind, placebo- and active-controlled study conducted at five sites between May 2016 to December 2016 in the United States among 407 patients recruited for an elective abdominoplasty surgery to determine whether oliceridine would provide rapid and superior acute postoperative analgesia compared to placebo with a more favorable safety and tolerability profile than morphine. The goal of this study was to confirm the previous oliceridine phase 2 findings where patients underwent either a bunionectomy or abdominoplasty. Similar analgesic efficacy was observed between the oliceridine treatment groups and morphine. Patients between the ages of 18-75 years were recruited to undergo an abdominoplasty procedure with no additional secondary procedures in the APOLLO-2 trial. Patients were eligible for randomization if they reported moderate to severe pain based on a categorical scale within four hours after surgery and if their pain was a score ≥5 on an NRS. 

Patients were randomized in equal ratios to double-blind IV treatment demand dose regimens of placebo, oliceridine 0.1 mg, oliceridine 0.35 mg, oliceridine 0.5 mg, or morphine 1 mg. They began administration with a clinician-administered IV fixed loading dose followed by the demand doses administered through a PCA device or clinician-administered blinded supplemental dose. The use of concomitant analgesics was prohibited for the most part. However, patients could receive protocol-specified open-label rescue pain medication with etodolac 200 mg every six hours as needed. These patients continued with the study medication unless both were inadequate. Prophylactic antiemetics and prophylactic supplemental oxygen were not permitted during the randomized treatment period.

The proportion of treatment responders at the 24-hour NRS assessment was significantly higher in all oliceridine cohorts (0.1 mg: 61%; 0.35 mg: 76.3%; 0.5 mg: 70%) compared to placebo (45.7%) and was similar to morphine’s efficacy (78.3%).

There was a dose-dependent increase of RSB across oliceridine regimens, but it was not statistically different from placebo (mean hours [standard deviation], oliceridine 0.1 mg: 0.43 [1.56]; oliceridine 0.35 mg: 1.48 [3.83]; oliceridine 0.5 mg: 1.59 [4.26] versus placebo: 0.60 [2.82]). However, this study showed that morphine was statistically significantly worse than placebo (1.72 [3.86]) indicating a difference in impact on RSB through the use of oliceridine. Ultimately, exploratory studies showed a lower incidence of respiratory safety events in the oliceridine regimens compared to morphine but were not statistically significant (oliceridine 0.35 mg: 21.5%, oliceridine 0.5 mg: 22.5% versus morphine 0.1 mg: 26.8%, P=0.20 and P=0.32). The cumulative duration of events was also not statistically significant. Therefore, no formal inferiority analyses were conducted, but exploratory studies did indicate that the 0.35 mg and 0.5 mg demand dose regimens were noninferior to morphine with the magnitude of pain relief comparable to morphine. The “perceptible pain relief” and “meaningful pain relief” did not show a statistical difference among the three oliceridine treatment groups and morphine. The active treatment regimens of oliceridine and morphine showed a notable decrease in rescue medication use. In fact, the use of these rescue medications was similar between the 0.35 mg and 0.5 mg oliceridine treatment groups and the morphine group. 

While the majority of the AEs reported were mild to moderate in intensity, five patients reported serious AEs. These were post-procedural hemorrhage, abdominal wall hematomas, syncope, and lethargy with the last two relating to oliceridine. The overall proportion of patients experiencing at least one AE was lowest with placebo at 78.3% and increased in a dose-dependent manner across the oliceridine groups (0.1 mg: 93.7%, 0.35 mg: 93.7%, 0.5 mg: 95%). The biggest proportion of patients experiencing at least one AE occurred in the morphine group at 97.6%. The most common AEs included nausea, vomiting, headache, and hypoxia. This study showed that fewer patients treated with oliceridine experienced nausea and vomiting compared to morphine. Previous studies have shown a reduced suppression of respiratory drive with oliceridine. The reduction in respiratory burden in this study was consistent with previous findings, but the differences between treatments using the composite outcome measure did not reach statistical significance. This may be due to the exclusion of patients at risk for these respiratory events. No deaths were reported in the trial. 

The clinical trials mentioned above are summarized in Table 3 for reference.

**Table 3:** Summary of Clinical Trials PCA, patient-controlled analgesia; AE, adverse event; NRS, numerical rating scale; IV, intravenous; GI, gastrointestinal; RSB, respiratory safety burden

ATHENA-1			
Author/Design	Intervention	Endpoint	Results
Bergese SD, Brzezinski M, Hammer GB, et al. [7] Phase 3, multi-center, open-label study evaluating the safety and effectiveness in patients with moderate to severe acute pain, warranting the use of a parenteral opioid (N=768)	Oliceridine through clinician-administered bolus dosing or through PCA	Number of patients that experienced a treatment-emergent AE	No statistical testing performed because observational study
		Mean change in the NRS pain score after 1 dose and end of treatment	Safety: 64% of patients reported at least one AE during the study (mild: 37%, moderate: 25%, severe: 2%)
			Effectiveness: mean change in NRS pain score (after 1 dose: −2.2 ± 2.3; end of treatment: −3.1 ± 3.1)
Conclusion: Oliceridine demonstrated a favorable safety profile in patients with opioid related complications for whom parenteral opioids were warranted when oliceridine was administered alone or as a component of multimodal analgesia.			
APOLLO-1			
Author/Design	Intervention	Endpoint	Results
Viscusi ER, Skobieranda F, Soergel DG, et al. [21] Phase 3, randomized, double-blind, placebo and active controlled study (N = 418)	Intervention administered through clinician-administered IV fixed loading dose followed by demand doses (Placebo, oliceridine 0.1 mg, oliceridine 0.35 mg, oliceridine 0.5 mg, morphine 1 mg)	Primary: proportion of treatment responders through 48 hours for oliceridine regimens and placebo	Efficacy: oliceridine showed statistical superiority compared to placebo among the proportion of treatment responders (P<0.0001)
		Secondary: composite measure of RSB and proportion of treatment responders vs. morphine	RSB: no statistically significant difference on composite outcome measure of RSB for the oliceridine treatment groups vs. morphine
			GI safety: AEs increased in a dose-dependent manner for oliceridine regimens compared to placebo (24.1%) and morphine (72.4%) (0.1 mg: 40.8%, 0.35 mg: 59.5%, 0.5 mg: 70.9%)
			GI safety: oliceridine regimens had significantly lower odds ratio for rescue antiemetic use vs. to morphine (P<0.05)
Conclusion: The findings demonstrate superior efficacy and safety of oliceridine for the management of moderate-to-severe pain following bunionectomy compared to the placebo. Benefit/risk profiles of IV oliceidine and morphine were evaluated by looking at efficacy, safety and tolerability data. The data suggested oliceridine can potentially be utilized as a treatment option for the management of patients experiencing moderate-to-severe acute pain.			
APOLLO -2			
Author/Design	Intervention	Endpoint	Results
Singla, NK, Skobieranda F, Soergel DG, et al. [22] Phase 3, multi-center, randomized, placebo- and active-controlled study to confirm the phase II study findings in patients recruited for elective abdominoplasty surgery (N=407)	Intervention administered through clinician-administered IV fixed loading dose followed by demand doses (Placebo, oliceridine 0.1 mg, oliceridine 0.35 mg, oliceridine 0.5 mg, morphine 1 mg)	Primary: proportion of treatment responders	Efficacy: proportion of treatment responders significantly higher in all oliceridine cohorts vs. placebo (0.1 mg: P=0.029, 0.35 mg: P<0.0001, 0.5 mg: P=0.0004)
		Secondary: magnitude of RSB	RSB: dose-dependent increase of RSB across oliceridine regimens vs. placebo but not statistically significant, but statistically significant difference between morphine and placebo (P<0.05)
		Secondary: treatment responders in oliceridine regimens vs. morphine	Safety: percent of patients experiencing at least one AE (placebo: 78.3%, oliceridine 0.1 mg: 89.6%, oliceridine 0.35 mg: 93.7%, oliceridine 0.5 mg: 95%, morphine: 97.6%)
		Secondary: pain intensity over time	
		Secondary: percentage of patients receiving rescue pain medication	
Conclusion: APOLLO-2 demonstrated the efficacy and safety of as needed oliceridine for the management of moderate to severe pain following abdominoplasty. Oliceridine showed statistically superior analgesia compared to placebo with a higher proportion of treatment responders in the 0.35- and 0.5-mg demand doses. The study showed a rapid onset of effect.			

Discussion

Surgeries such as bunionectomies and abdominoplasties may require management of postoperative complications including pain. The management of preoperative, intraoperative, and postoperative pain is continuously evolving. According to the clinical practice guidelines from the American Pain Society, the treatment of postoperative pain should be individually tailored to a patient’s specific needs on the basis of adequate pain relief and the presence of AEs [2]. With the high percentage of patients experiencing moderate to severe postoperative pain, opioids are used especially during the first 24 hours after surgery. Intravenous opioids have not shown superiority to oral administration and therefore, in the initial pain management, short-acting oral opioids are preferred for moderate to severe analgesia [2]. While IV morphine may not seem an appropriate choice for the treatment of moderate to severe pain due to the high risk of serious AEs, it is still widely used and is the main opioid assessed in many studies of immediate postoperative pain management due to its high efficacy and potency [23]. Patient-related risk factors may limit their ability to handle these AEs. OIRD is the most serious AE and the biggest driver for the increased deaths associated with opioid use. It can cause life-threatening respiratory and cardiopulmonary arrest. While exploratory studies showed that RSB was more favorable in the oliceridine group compared to morphine, it was never directly studied but solely based on clinicians’ subjectivity [24]. According to a survey of 501 physicians treating postoperative pain, the highest reported unmet need for postoperative pain management is an IV opioid with fewer side effects [25]. Oliceridine demonstrates the potential to address this gap in treatment as seen through the phase 3 clinical trials discussed earlier. 

The approval of oliceridine highlights the impact of the opioid epidemic in America. This crisis has impacted the lives of many throughout the decades. Since 1999, there have been three waves of opioid overdose deaths each involving different opioids, including prescription or illicit opioids [26]. The only way to combat this opioid epidemic is through the collaboration of many different groups, including medical personnel. This can improve the ways opioids are prescribed and reduce the number of people who misuse or overdose from these drugs. Since 2011, opioids have been prescribed less as a whole nationally in response to the opioid epidemic. However, the need for surgeries and anesthesia has increased and will continue to increase over the next decade [27]. This trend has correlated with the increase in opioid prescribing to treat patients postoperatively and there has been an increase of 70% in the average total of morphine equivalents prescribed for postoperative pain leading to increased opioid-related morbidity and mortality [5].

The opioid epidemic has shed light on the importance of alleviating acute pain. Factors, such as age, gender, income, education, and type of surgery impact one’s pain vulnerability. The United States Centers for Disease Control and Prevention (CDC) has developed guidelines to work towards combating the overuse of opioids in chronic pain, but pain management guidelines have done very little to address the issue of the increased prescribing in the acute setting [6]. The answer to the opioid epidemic is not to completely abandon the use of opioids as they play a major role in the management of moderate to severe pain; it is to control the use and monitor for the addiction.

## Conclusions

Future outlook

Through the ATHENA-1, APOLLO-1, and APOLLO-2 trials, oliceridine has demonstrated potential as an opioid with a new mechanism of action showing superior efficacy results compared to placebo, comparable efficacy results to morphine, and a favorable safety profile, but head to head studies comparing oliceridine and morphine are lacking. The exploratory studies were mainly focused on the impact of oliceridine on specific ORAEs, but as a whole, the phase 3 clinical trials conducted for oliceridine did not account for specific imbalances. For example, they predominantly enrolled females, failing to represent the findings across both genders. Additionally, prophylactic antiemetics were prohibited in APOLLO-1 and APOLLO-2. As a result, it would be difficult to predict the occurrence of postoperative nausea and vomiting with oliceridine compared to conventional opioids. As stated earlier, these ORAEs can be prevalent in younger patients and, therefore, future studies with oliceridine should be conducted in this population. 

Ultimately, oliceridine provides a new alternative for clinicians due to its rapid onset of action, potent pain relief, and favorable safety profile. While a major aspect of the opioid epidemic is the addiction potential of these opioids associated with long-term use, the potential harm of ORAEs associated with short-term use is a growing area of concern. The approval of oliceridine is a stepping stone in the fight against the opioid epidemic because it paved the path towards finding the balance between adequate management and minimized AEs. The reported safety profile of oliceridine is similar to that of the current pain management options in terms of common side effects, but the selective pathway of this G protein-biased μ-opioid receptor agonist presents a new alternative to clinicians requiring IV analgesia in patients at higher risk for these ORAEs.

## References

[1] Gupta A, Kaur K, Sharma S, Goyal S, Arora S, Murthy RS (2010). Clinical aspects of acute post-operative pain management & its assessment. J Adv Pharm Technol Res.

[2] Chou R, Gordon DB, de Leon-Casasola OA (2016). Management of postoperative pain: a clinical practice guideline from the American Pain Society, the American Society of Regional Anesthesia and Pain Medicine, and the American Society of Anesthesiologists' Committee on Regional Anesthesia, Executive Committee, and Administrative Council. J Pain.

[3] Stephens J, Laskin B, Pashos C, Peña B, Wong J (2003). The burden of acute postoperative pain and the potential role of the COX-2-specific inhibitors. Rheumatology (Oxford).

[4] Gan TJ (2017). Poorly controlled postoperative pain: prevalence, consequences, and prevention. J Pain Res.

[5] Upp LA, Waljee JF (2020). The opioid epidemic. Clin Plast Surg.

[6] Williamson KJ, Stram ML (2019). The epidemiology of inadequate control of acute pain. Pain.

[7] Bergese SD, Brzezinski M, Hammer GB (2019). ATHENA: a phase 3, open-label study of the safety and effectiveness of oliceridine (TRV130), a G-protein selective agonist at the µ-opioid receptor, in patients with moderate to severe acute pain requiring parenteral opioid therapy. J Pain Res.

[8] Chung CP, Callahan ST, Cooper WO (2018). Outpatient opioid prescriptions for children and opioid-related adverse events. Pediatrics.

[9] Garimella V, Cellini C (2013). Postoperative pain control. Clin Colon Rectal Surg.

[10] Harricharan S, Frey N (2018). Intravenous Acetaminophen for the Management of Short-Term Post-Operative Pain: A Review of Clinical Effectiveness and Cost-Effectiveness [Internet]. https://www.ncbi.nlm.nih.gov/books/NBK538274/.

[11] Jibril F, Sharaby S, Mohamed A, Wilby KJ (2015). Intravenous versus oral acetaminophen for pain: systematic review of current evidence to support clinical decision-making. Can J Hosp Pharm.

[12] Gerriets V, Anderson J, Nappe TM (2021). Acetaminophen. https://europepmc.org/article/nbk/nbk482369.

[13] Carter JA, Black LK, Sharma D, Bhagnani T, Jahr JS (2020). Efficacy of non-opioid analgesics to control postoperative pain: a network meta-analysis. BMC Anesthesiol.

[14] Smith HS (2011). Perioperative intravenous acetaminophen and NSAIDs. Pain Med.

[15] Ghlichloo I, Gerriets V (2021). Nonsteroidal anti-inflammatory drugs (NSAIDs). StatPearls [Internet].

[16] Bell RF, Kalso EA (2018). Ketamine for pain management. Pain Rep.

[17] (2021). Ketamine hydrochloride- ketamine hydrochloride injection, solution, concentrate. http://labeling.pfizer.com/ShowLabeling.aspx?id=4485.

[18] Kung J, Meisner RC, Berg S, Ellis DB (2021). Ketamine: a review of an established yet often underappreciated medication. Newsletter J APSF.

[19] (2021). FDA approves new opioid for intravenous use in hospitals, other controlled clinical settings. https://www.fda.gov/news-events/press-announcements/fda-approves-new-opioid-intravenous-use-hospitals-other-controlled-clinical-settings.

[20] (2021). Olinvyk (oliceridine) injection, for intravenous use, CII. Chesterbrook, PA: Trevena, Inc.

[21] Viscusi ER, Skobieranda F, Soergel DG, Cook E, Burt DA, Singla N (2019). APOLLO-1: a randomized placebo and active-controlled phase III study investigating oliceridine (TRV130), a G protein-biased ligand at the µ-opioid receptor, for management of moderate-to-severe acute pain following bunionectomy. J Pain Res.

[22] Singla NK, Skobieranda F, Soergel DG, Salamea M, Burt DA, Demitrack MA, Viscusi ER (2019). APOLLO-2: a randomized, placebo and active-controlled phase iii study investigating oliceridine (TRV130), a G protein-biased ligand at the μ-opioid receptor, for management of moderate to severe acute pain following abdominoplasty. Pain Pract.

[23] Aubrun F, Mazoit JX, Riou B (2012). Postoperative intravenous morphine titration. Br J Anaesth.

[24] Tan HS, Habib AS (2021). Oliceridine: a novel drug for the management of moderate to severe acute pain - a review of current evidence. J Pain Res.

[25] Gan TJ, Epstein RS, Leone-Perkins ML, Salimi T, Iqbal SU, Whang PG (2018). Practice patterns and treatment challenges in acute postoperative pain management: a survey of practicing physicians. Pain Ther.

[26] (2021). Understanding the epidemic. https://www.cdc.gov/opioids/basics/epidemic.html.

[27] Meara JG, Leather AJM, Hagander L (2015). Global surgery 2030: evidence and solutions for achieving health, welfare, and economic development. Lancet.

